# Did Silencing Suppression Counter-Defensive Strategy Contribute to Origin and Evolution of the Triple Gene Block Coding for Plant Virus Movement Proteins?

**DOI:** 10.3389/fpls.2012.00136

**Published:** 2012-07-06

**Authors:** Sergey Y. Morozov, Andrey G. Solovyev

**Affiliations:** ^1^Belozersky Institute of Physico-Chemical Biology, Moscow State UniversityMoscow, Russia

Comparison of gene silencing in tissues and whole organisms shows intriguing similarities between plants and animals (Cohen and Xiong, 2011; Hyun et al., 2011; Jose et al., 2011; Melnyk et al., 2011; Molnar et al., 2011) despite that they are very different from each other in many aspects related to the cell-to-cell communications (Ritzenthaler, 2011). Interestingly, one of the shared mechanisms is the reprogramming of intracellular silencing pathways and intercellular communications during development of virus infections. As a part of their counter-defensive strategy, viruses encode silencing suppressors to inhibit various stages of the silencing process. These suppressors are diverse in sequence and structure and act via different molecular mechanisms including, particularly, blockage of intercellular and systemic spread of mobile small interfering RNAs (siRNAs; Li and Ding, 2006; Bivalkar-Mehla et al., 2011; Burgyán and Havelda, 2011; Shimura and Pantaleo, 2011; Song et al., 2011). Importantly, plant, insect, and animal virus suppressors can substitute for each other in different eukaryotic model systems (Schnettler et al., 2008; Jing et al., 2011; Maliogka et al., 2012; Zhu et al., 2012). Many viral proteins that in the past were characterized as proteins involved in systemic plant invasion are now known to be suppressors of gene silencing. For example, *tombusvirus* P19 blocks the intercellular movement of the silencing signal by binding DCL4-dependent 21-nt siRNA. *Cucumovirus* 2b protein inhibits the systemic movement of RNA silencing by either binding dsRNA/siRNA or inhibiting the slicer activity of AGO1. *Potato virus X* P25 protein also inhibits the systemic movement of RNA silencing (Li and Ding, 2006; Burgyán and Havelda, 2011; Shimura and Pantaleo, 2011). Direct link between the viral suppressor activity and the ability of virus to move cell-to-cell and long-distance is further strengthened by the discovery of plant movement proteins (MPs) acting also as silencing suppressors (Bayne et al., 1995; Voinnet et al., 1999; Yaegashi et al., 2007; Powers et al., 2008; Lim et al., 2010; Wu et al., 2010; Senshu et al., 2011; Renovell et al., 2012). On the other hand, it has been shown that the MPs of certain viruses act as viral enhancers of RNA silencing by promoting the propagation of RNA silencing from cell to cell (Vogler et al., 2008; Zhou et al., 2008; Lacombe et al., 2010; Amari et al., 2012).

Unlike *Tobacco mosaic virus* and many other viruses having a single MP gene, the genomes of a number plant virus genera encode a triple gene block (TGB), a specialized evolutionarily conserved gene module involved in the movement of viruses. The TGB-based transport system exploits the co-ordinated action of three polypeptides to deliver viral genomes to plasmodesmata (PD) and to accomplish virus entry into neighboring cells. TGB-encoded proteins are referred to as TGB1, TGB2, and TGB3 (Morozov and Solovyev, 2003; Verchot-Lubicz et al., 2010). We present here a hypothetical model of how interaction of plant viruses with the silencing machinery may contribute to the TGB origin and evolution during adaptation of viruses to land plant hosts. The hypothesis was stimulated by the previous evidence indicating that the suppression of silencing by TGB1 protein encoded by potex- and carlaviruses is not sufficient to allow virus movement between cells, and there must be another function of this protein independent of silencing but required for cell-to-cell movement (Bayne et al., 1995; Lim et al., 2010; Senshu et al., 2011). Similarly to TGB-containing viruses, suppression of local RNA silencing is not sufficient to promote cell-to-cell movement of *Turnip crinkle virus* (Shi et al., 2009).

In principle, there are three distinct scenarios for the evolution of viruses: first, evolution from a common ancestral virus accompanying the divergence of host taxonomic groups; second, horizontal transfer of viruses and their genomic elements; third, parallel origin from related genetic elements (Dolja and Koonin, 2011). If we take first principle as the main evolutionary flow for plant plus-RNA viruses, algae (especially those included into the kingdom Viridiplantae) should be considered as hosts for precursors of land plant viruses. It is currently well documented that green algae possess many components that are assumed to be involved in RNA silencing mechanisms in other better studied eukaryotes (Ahn et al., 2010; Cerutti et al., 2011). Correspondingly, algal viruses should have evolved to acquire silencing suppressors making possible establishing successful infection. However, most green algae-infecting viruses sequenced so far (classified in the virus family Phycodnaviridae) are among the largest known DNA viruses (Weynberg et al., 2011; Van Etten and Dunigan, 2012). We are still in an initial phase of understanding in algal RNA virology and, as new genomic technologies become more widely used in this field, we will see an exponential rise in number of sequenced plus-RNA algal virus genomes. Metagenomics provide a way to bypass the difficulty of obtaining genomic information about viruses that are hard to retrieve in pure culture. There are large datasets of metaviriomes, and they often can be assembled into nearly complete genomic RNAs to study the enormous diversity of the genes of viruses and to help in the annotation of viral ORFs (Kristensen et al., 2010).

**Figure 1:**
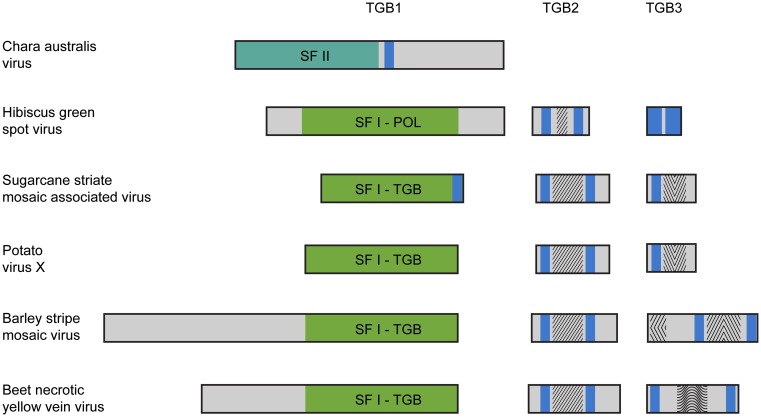
**Organization of triple gene block (TGB)-encoded proteins of some plant viruses**. Boxes schematically represent open reading frames. It should be noted that in fact the TGB2 coding sequence overlaps the 3′ end of TGB1 gene, and the TGB3 ORF overlaps the 3′ end of TGB2 gene (Morozov and Solovyev, 2003). Helicase domains are indicated in the green boxes (SF-I) and dark green box (SF-II). Blue boxes represent the hydrophobic segments found in TGB proteins. Conserved sequence signatures in hydrophilic segments of TGB2 and TGB3 proteins are indicated by different shading within the boxes. In all studied TGBs, the TGB2 proteins have a highly conserved signature in the central part of their sequence. Note that the *Hibiscus green spot virus* TGB2 protein includes a shortened version of the TGB2 conserved signature. The TGB3 proteins are diverse in different virus groups. In *Potato virus X* and *Sugarcane striate mosaic virus* TGB3 proteins, a common signature is located downstream of their single transmembrane domain. This signature is characteristic for the TGB3 proteins in viruses of genera *Potexvirus*, *Allexivirus*, *Mandarivirus*, *Carlavirus*, and *Foveavirus*. In the *Barley stripe mosaic virus*, TGB3 has two unique signatures typical for viruses of the genera *Hordeivirus* and *Pomovirus* located in the N-terminal and central hydrophilic sequence segments. In the *Beet necrotic yellow vein virus* (BNYVV) TGB3, two transmembrane domains are located close to the protein termini, and there is a conserved signature characteristic of the genus *Benyvirus* only, which is located in the central protein part. The *Hibiscus green spot virus* TGB3 protein contains extremely short central hydrophilic region.

Until now we have only single example of well-characterized plus-RNA virus from algae closely related to land plants. This is *Chara australis virus* (CAV; Gibbs et al., 2011), the largest encoded protein of which shows the relationship with RNA polymerases of benyviruses, while the coat protein – with the coat protein of tobamoviruses, thus reflecting the ancient sister relationship between hosts of these viruses, charophytes and land plants. Two additional CAV ORFs code for non-replicative RNA helicase and a protein of unknown function. Importantly, this CAV helicase is related to CI helicase (SF-II) of Ipomoviruses (family Potyviridae; Figure 1), which is involved in cell-to-cell movement in addition to replication (Wei et al., 2010). Emergence by CAV genome a second SF-II helicase in addition to unrelated replicative SF-I helicase is intriguing assuming the newly discovered role of cell SF-II helicases in the RNA interference and antiviral host defense (Ulvila et al., 2010). Organization of multicellular charophytes is rather close to land plants and they contain PD which are morphologically similar to higher plant PD (Brecknock et al., 2011). Thus we can propose that plus-RNA viruses of unicellular algae in the course of transition of hosts to multicellularity may evolve additional RNA helicase genes (either by shuffling with distantly related viruses or by duplication of helicase domain in own replicase) required for virus genome spread over the plant organism (Figure 1).

It is known that plant virus RNA replicases may significantly contribute to virus silencing suppressor activity (Ding et al., 2004; Mine et al., 2010), and RNA helicase domain (Koonin and Dolja, 1993) may play important separate role in this activity (Wang et al., 2012). Similarly, replicative DNA helicases of single-stranded plant DNA viruses may be involved in silencing suppression (Nawaz-Ul-Rehman et al., 2010). The TGB is found in viruses of the “alpha-like” supergroup only (families Virgaviridae, Alphaflexiviridae, Betaflexiviridae, and genus *Benyvirus*; Koonin and Dolja, 1993; Adams et al., 2009; Verchot-Lubicz et al., 2010), suggesting a specific co-adaptation between replication and movement genes. TGB1 contains an NTPase/helicase sequence domain that is related to the replicative helicases of alpha-like viruses and belongs to helicases of superfamily I (SF-I; Koonin and Dolja, 1993). Phylogenetic analysis of the NTPase/helicase sequences reveals clustering of the TGB1 proteins into two major groups (Wong et al., 1998), corresponding to filamentous viruses (genera *Potexvirus*, *Carlavirus*, *Foveavirus*, and *Allexivirus*), which also exploit this protein as silencing suppressor (Bayne et al., 1995; Lim et al., 2010; Senshu et al., 2011), and rod-shaped viruses (genera *Hordeivirus*, *Benyvirus*, *Pomovirus*, and *Pecluvirus*). Furthermore, the molecular masses of TGB1 in filamentous viruses range from 24 to 26 kDa and the NTPase/helicase domain comprises the entire sequence, whereas TGBp1s of rod-shaped viruses are substantially larger – from 39 to 63 kDa – and contain the additional long N-terminal domains (Morozov and Solovyev, 2003). Obviously, the emergence of replicative RNA helicases in viral RNA genomes is an early evolutionary step since, although eukaryotic RNA viruses with genomes shorter than 6 kB usually do not code for RNA helicases, the larger RNA viruses of diverse eukaryotes all code for RNA helicases (Koonin and Dolja, 1993). Assuming appearance of helicase-related silencing suppressors by gene duplication or shuffling and its further possible evolution resulting in gaining the MP function, it can be hypothesized that precursors of land plant RNA viruses could evolve to share two specialized functions (silencing suppression and movement) in a single replication-related gene, which has lost its replication function.

Further evolution of such virus genomes may result in origination of the TGB. The main driving force of this evolutionary step could be dependence of helicase activity on the membrane binding. Indeed, positive-sense RNA viruses are known to exploit host cell membranes to facilitate RNA replication. Formation of these replication sites often involves virus-induced membrane synthesis, changes in fatty acid metabolism, and viral recruitment of cellular factors to membrane subcellular domains serving as a physical scaffold for replication complexes (Sasvari and Nagy, 2010; Stapleford and Miller, 2010). Autonomized helicase domains, although have lost their replication function, may still require membrane-based microenvironments for their silencing and/or movement activity. This requirement may be achieved at first by evolving protein sequences with the hydrophobic membrane-bound segment(s) (Figure 1). Indeed, we revealed that potex-like TGB1 protein of *Sugarcane striate mosaic virus* possesses confidently predicted transmembrane segment in the extreme C-terminal region (Figure 1). Moreover, this prediction analysis revealed a membrane-bound segment in the central region of the CAV non-replicative helicase (Figure 1). Although these examples represent only rare cases of occurrence of hydrophobic membrane segments in viral helicase proteins, it may reflect real events happened early in evolution of autonomized helicases. Further (or alternative) event to achieve membrane binding of autonomized helicases possibly included evolving specialized membrane proteins capable of interacting with both cell membranes and helicase proteins. This evolutionary process probably caused the origination of small membrane-bound proteins TGB2 and TGB3 (Morozov and Solovyev, 2003; Verchot-Lubicz et al., 2010; Figure 1). The TGB2 gene could have emerged by further autonomization of the C-terminal transmembrane domain of non-replicative helicase resulted, for example, from a frame-shift mutation bringing the future TGB2 sequence into another reading frame. The TGB3 gene, which significantly overlaps the TGB2 gene, was predicted to appear by overprinting, in which an existing coding sequence is becoming to be translated in two distinct reading frames (Keese and Gibbs, 1992; Rancurel et al., 2009). Thus, these TGB3 genes may represent more recent evolutionary event in the TGB formation on the way to viral adaptation to the RNA trafficking pathways of the hosts. Possible independent origin of small TGB genes in some virus families explains the structural and functional diversity of TGB3 proteins identified among viruses sequenced so far (Morozov and Solovyev, 2003; Rancurel et al., 2009; Figure 1). This is well illustrated by the very recent example of *Hibiscus green spot virus* (HGSV) which codes for polymerase related to that of family Virgaviridae (Melzer et al., 2012). However, HGSV TGB1 helicase is only very distantly related to other TGB1 proteins and shows more similarity to replicative helicases of genus *Benyvirus* representing a distinct group within the alpha-like supergroup of positive-strand RNA viruses (Koonin and Dolja, 1993). TGB3 protein of HGSV contains two long hydrophobic segments and shows no significant similarity to any of three known groups of TGB3 proteins (hordei-, beny-, and potex-like TGB3’s; Figure 1). Interestingly, despite the fact that TGB2 is most conserved among proteins of hordei-, beny-, and potex-like TGB's (Morozov and Solovyev, 2003), the HGSV protein has the central hydrophilic segment which is only distantly related to other TGB2’s (Figure 1).

In conclusion, new data on interrelation of RNA silencing and virus movement and, on the other hand, recently published sequences of new TGB-containing viruses allowed us to put forward the hypothesis of a three-step TGB origin in virus evolution. These steps include autonomization of a second virus RNA helicase initially possessing the function of silencing suppression, gaining the virus movement function by this protein, and acquisition of accessory membrane proteins.
